# Reprogramming of Energy Metabolism in Response to Radiotherapy in Head and Neck Squamous Cell Carcinoma

**DOI:** 10.3390/cancers11020182

**Published:** 2019-02-05

**Authors:** Alfredo Cruz-Gregorio, Imelda Martínez-Ramírez, José Pedraza-Chaverri, Marcela Lizano

**Affiliations:** 1 Unidad de Investigación Biomédica en Cáncer, Instituto Nacional de Cancerología, México/Instituto de Investigaciones Biomédicas, Universidad Nacional Autónoma de México. San Fernando No. 22, Col. Sección XVI, Tlalpan, Ciudad de México 14080, México; cruzgalfredo@gmail.com (A.C.-G); immara02@yahoo.com.mx (I.M.-R); 2 Departamento de Biología, Facultad de Química, Universidad Nacional Autónoma de México, Ciudad Universitaria, Ciudad de México 04510, México; 3 Departamento de Medicina Genómica y Toxicología Ambiental, Instituto de Investigaciones Biomédicas, Universidad Nacional Autónoma de México, Ciudad Universitaria, Ciudad de México 04510, México

**Keywords:** HNSCC, HR-HPV, radiotherapy, reprogramming of energy metabolism, metabolic therapy, oxidative stress

## Abstract

Head and neck cancer (HNC) is the sixth cause of cancer-related death worldwide. Head and neck squamous cells carcinoma (HNSCC) is the most frequent subtype of HNC. The development of HNSCC is associated to alcohol consumption, smoking or infection by high-risk human Papillomavirus (HR-HPV). Although the incidence of cancers associated with alcohol and tobacco has diminished, HNSCC associated with HR-HPV has significantly increased in recent years. However, HPV-positive HNSCC responds well to treatment, which includes surgery followed by radiation or chemoradiation therapy. Radiation therapy (RT) is based on ionizing radiation (IR) changing cell physiology. IR can directly interact with deoxyribonucleic acid (DNA) or produce reactive oxygen and nitrogen species (RONS), provoking DNA damage. When DNA damage is not repaired, programmed cell death (apoptosis and/or autophagy) is induced. However, cancer cells can acquire resistance to IR avoiding cell death, where reprogramming of energy metabolism has a critical role and is intimately connected with hypoxia, mitochondrial physiology, oxidative stress (OS) and autophagy. This review is focused on the reprogramming of energy metabolism in response to RT in HPV-positive and HPV-negative HNSCC, showing their differences in cellular metabolism management and the probable direction of treatments for each subtype of HNSCC.

## 1. Introduction

Head and neck cancer (HNC) represents the sixth cause of cancer-related death worldwide, affecting mainly the male sex [1]. Head and neck squamous cell carcinoma (HNSCC) is the most abundant subtype of HNC. These malignancies are associated to alcohol consumption and smoking; however, infection by high-risk human Papillomavirus (HR-HPV) has been implicated in the development of HNSCC. The proportion of HNSCC associated with HR-HPV has increased from 16.3% during 1984 to 1989 to 71.7% in the period of 2000–04 in the United States [2]. However, it has been shown that the HNSCC cases positive to HPV have a better response to radiation treatment in comparison with HPV-negative HNSCC cases [3]. 

Persistent infection with HR-HPV is the main risk factor for HPV-positive HNSCC, where HPV-16 is the most prevalent type [4]. The development of cancer is associated to the constant expression of E6 and E7 HPV oncoproteins, which modulate numerous cellular signaling pathways, resulting in immortalization and transformation [5]. A key feature in cancer development is the viral genome integration into host genome, which commonly leads to the breaking of the viral E2 open reading frame, promoting the overexpression of E6 and E7 oncogenes [6]. The process of integration is associated to different facts that induce deoxyribonucleic acid (DNA) breaks; for example: rupture of chromosomal fragile sites [7], decrease in DNA repair machinery activity [8], replication stress [9] or oxidative stress (OS) [10].

**Figure 1:**
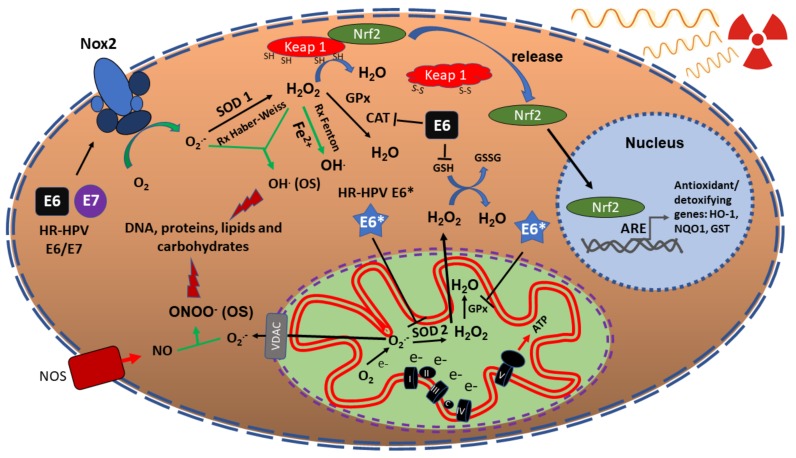
Redox state in irradiated HNSCC cells and the effect of HPV oncoproteins. HNSCC cells regulate oxidative stress induced by IR through the overexpression of Nrf2, which in turn activates genes such as catalase, superoxide dismutase (SOD2) and glutathione peroxidase (GPx), associated to antioxidant processes. On the other hand, the HR-HPV E6 protein induce OS, which decreases the GSH/GSSG rate, the levels of catalase and its activity [44]. E6* decreases SOD2 and GPx, provoking OS and consequently, DNA damage [45]. E6/E7 activate Nox2, inducing OS and DNA damage [46]. Therefore, altogether, HPV oncoproteins could sensitize HNSCC cells to radiation therapy. Nicotinamide adenine dinucleotide phosphate (NADPH) oxidase 2 (Nox2), superoxide dismutase (SOD), heme oxygenase (HO-1), NAD(P)H:quinone oxidoreductase 1 (NQO1), glutathione S-transferase (GST), glutathione peroxidase (GPx), catalase (CAT), glutathione (GSH), glutathione disulfide (GSSG), voltage-dependent anion channels (VDAC), peroxynitrite (ONOO^−^), nitric oxide (NO), superoxide anion (O_2_^.−^), hydrogen peroxide (H_2_O_2_), hydroxyl radical (OH^.^), high-risk human Papillomavirus (HR-HPV), nuclear factor (erythroid-derived 2)-like 2 (Nrf2), antioxidant response elements (ARE), nitric oxide synthase (NOS). The red rays indicate the damage to DNA, protein, carbohydrates and lipids.

Radiotherapy (RT) used for tumour elimination is mainly based on ionizing radiation (IR). IR consists in electromagnetic radiation such as γ-rays or χ-rays, which carries enough energy to liberate electrons from atoms or molecules, ionizing them [11]. IR can interact with DNA provoking DNA double-strand breaks (DSBs), single-strand breaks (SSBs) or can induce the production of reactive oxygen and nitrogen species (RONS), such as superoxide anion (O_2_^.−^), hydrogen peroxide (H_2_O_2_), hydroxyl radical (OH^.^) and peroxynitrite (ONOO^−^), among others [12]. These RONS are neutralized by different cellular antioxidant enzymes such as: superoxide dismutases (SODs), catalase (CAT), glutathione peroxidase (GPx), peroxiredoxin (Prxs), and thioredoxine reductase (TrxR); and non-enzymatic antioxidants including: glutathione (GSH) and vitamins A, C, or E (Figure 1). However, if RONS overpass the antioxidant effects, a cellular oxidant condition appears called OS, where cellular molecules such as DNA, proteins, or lipids are oxidized and damaged, leading to cell death [13]. Thus, IR induces cancer cell death directly and indirectly through DNA damage and OS. Nevertheless, after cancer cells are exposed to IR, numerous cell processes are activated, where resistance to IR can be induced, avoiding cell death. For example, cancer cells can increase the DNA damage response (DDR) rate, which involves the activation of different signaling pathways associated to the DSB repairing, decreasing the DNA damage induced by IR. One of those signaling pathways is the epidermal growth factor receptor (EGFR)-phosphatidylinositol 3-kinase (PI3K)/protein kinasa B (Akt) pathway, where DNA-dependent protein kinase (DNA-PK), a molecule required for DNA repair via non-homologous end joining (NHEJ), is activated [14]. 

IR activates the ataxia telangiectasia mutated (ATM)-checkpoint kinase 2 (Chk2) pathway, which detects DSB and activates p53, breast cancer (BRCA) protein, p95/NBS1 and CtlP, inducing cell cycle arrest and DSB repair [15]. IR also activates the ATR-Chk1 pathway [16], which senses DNA SSBs, which in turn activates p38 mitogen-activated protein kinases (MAPKs), promoting DNA repair through the activation of the χ-ray repair cross-complementing protein 1 (XRCC1) [17]. Moreover, IR induces the overexpression of sirtuin 1 (SIRT1), which then activates the apurinic/apyrimidinic endodeoxyribonuclease (APEX1), stimulating the DNA base excision repair (BER) pathway [18,19]. 

Additionally, IR deregulates cell adhesion molecules such as E-cadherin, N-cadherin, Wnt1-inducible signaling pathway protein-1 (WISP1), vimentin, twist-related protein 1 (TWIST), SNAIL1, fibronectin and vitronectin (VTN), which favour the epithelial mesenchymal transition (EMT) [20,21,22,23]. It is noteworthy that when DDR and EMT are induced in the tumour cells, the tumour microenvironment (TME) is also affected, playing a critical role in the acquisition of radioresistance. For example, IR induces damage and death of the endothelial cells that surround tumour cells, which increases cytokines and chemokines levels, initiating an inflammatory response [24]. Chronic inflammation induces fibrosis in the endothelial cells, characterized by the repair and remodelling of the tissue, where molecules such as integrins, transforming growth factor-β (TGFβ) and connective tissue growth factor (CTGF) are secreted, which provoke tumour cell survival [25]. 

The cancer-associated fibroblasts (CAF) are key cells in the TME, that when exposed to IR, release growth factors such as TGF-β, extracellular matrix (ECM) modulators and cytokines, favouring tumour cell growth. IR reduces vascular diameter, activating hypoxia-inducible factor 1α (HIF1α) and vascular endothelial growth factor (VEGF), provoking the release of chemokine (C-X-C motif) ligand 12 (CXCL12), which altogether protect tumour cells from cellular stress and death. Therefore, IR affects the vasculature, immune system and stroma, which could induce hypoxia and radioresistance [26].

A critical cellular condition in the induction of radioresistance is the reprogramming of energy metabolism, which is a cellular condition favoured in cancer cells, where energy production in the form of ATP is mainly obtained through glycolysis (Warburg effect) instead of oxidative phosphorylation (OXPHOS), even in presence of oxygen and with mitochondrial optimal function [27]. This hallmark of cancer can be presented in different fashions among the different cancers, where in certain conditions cells use either glycolysis or OXPHOS to obtain energy. Nevertheless, the use of glycolysis or OXPHOS could change in response to IR. Therefore, this review is focused on the mechanisms involved in the reprogramming of energy metabolism during radiotherapy response in HPV-negative and HPV-positive HNSCC.

## 2. Genetic Alterations in HPV-Positive and HPV-Negative HNSCC

HPV-positive and -negative HNSCC present differences in etiology, molecular properties, epidemiology, genetic alterations (mutations and copy number aberrations) and in their response to treatment [28,29,30]. Some studies report that the mutation rate is higher in HPV-negative HNSCC in relation to HPV-positive cases [31,32]. In contrast, other studies show that the mutation rate is not significantly different between both groups, although the genomic aberration profiles are different [33,34]. For instance, HPV-negative HNSCC has a higher number of mutations in TP53, CDKN2A, MLL2, CUL3, NSD1, FGFR1, DDR2, EGFR, FGFR2/3, EPHA2, PIK3CA and NOTCH genes; while HPV-positive cases present mutations in genes such as DDX3X, FGFR2/3, PIK3CA, KRAS and MLL2/3 [33]. Interestingly, some mutations can coincide in both groups; however, the mutation rate in a set of genes is different in relation to the HPV status. For example, PIK3CA, DDR2 or NF-kB genes are significantly mutated in HPV-positive HNSCC, which are associated to the activation of glutamine and lipid metabolisms and mitochondrial respiration, key processes involved in the development of these tumours [35,36,37]. On the contrary, mutations found in HPV-negative HNSCC cases inactivate the function of tumour suppressors such as p53, favouring glycolysis; since when p53 is functionally active, it induces the inhibition of glycolysis mainly through the activation of the protein TP53-induced glycolysis and apoptosis regulator (TIGAR) and the repression of glucose transporter 1 and 4 (GLUT1 and GLUT4) [38]. Therefore, the loss of p53 probably contributes to the induction of the Warburg effect instead of OXPHOS in order to obtain energy in the form of ATP. 

## 3. Radiosensitivity in HPV-Positive HNSCC

Alcohol consumption, smoking, and infection with HR-HPV are the main etiological factors associated to HNSCC [39,40]. Regardless of the etiologic factor, the treatment regimen for HNSCC is usually the same; nevertheless, HNSCC can be divided into two subgroups: HPV-positive and HPV-negative, having mutually exclusive features. HPV-positive HNSCC patients have a better prognosis, whose HNSCC tumours present lower p53 mutation rate and have a better response to IR than HPV-negative tumours [41]. 

In HPV-positive HNSCC, the residual p53, when it is not degraded by the HPV E6 oncoprotein, can be activated after IR, inducing cell cycle arrest and apoptosis [42]. It has also been demonstrated that the capacity of repair of DSB is impaired in HPV-positive HNSCC [43]. 

Previously, we showed that HPV16 and HPV18 early-expressed proteins E1, E2, E6 and E7, differentially modulate the cellular redox state [44]. The co-expression of E1/E2 decreased the relation of reduced vs oxidized GSH (GSH/glutathione disulfide [GSSG]) and also decreased the expression and activity of SOD1/2, increasing RONS levels, OS and DSB. It was also demonstrated that the E6 oncoprotein increased RONS levels, OS and DSB, reducing the rate of GSH/GSSG, catalase expression and its activity, while E7 decreased RONS production due to the increase in GSH and catalase levels and its activity [44]. On the other hand, it has been shown that E6*, an isoform of E6, decreased the levels of SOD2 and GPx proteins, inducing reactive oxygen species (ROS) production (Figure 1) [45]. Those works suggest that the presence of different levels of E6 and E7 proteins in particular cellular contexts could influence the different phenotypes of HPV-positive tumours. Therefore, OS induced by HR-HPV could synergize with the OS induced by IR, eliciting the radiosensitivity observed in HPV-positive HNSCC. 

OS associated to ROS deregulation is harmful and favours cell death. For example, H_2_O_2_ (even O_2_^.−^) reacts in the Fenton reaction to induce OH^.^ production, which in turn induces DNA damage; if DNA is not repaired, cellular death via apoptosis is induced [47]. However, ROS are also necessary for cell proliferation. Such is the case of H_2_O_2_ that can oxidize cysteines inducing disulphide bonds in proteins such as growth factor receptors (GFR), which are then activated promoting cellular proliferation [48]. The concentration of ROS establishes either cell death or proliferation. Therefore, ROS balance is essential to maintain cellular homeostasis. Cancer cells can augment the DDR rate and even control elevated ROS concentrations by inducing the enzymatic antioxidant expression (SOD and CAT, among others) or by increasing levels of non-enzymatic antioxidants, such as GSH or nicotinamide adenine dinucleotide phosphate (NADPH). NADPH indirectly regulates ROS production, since NADPH is a cofactor of different antioxidant enzymes such as the thioredoxin reductase (TrxR) and glutathione reductase (GR), which in turn are associated to the reduction of peroxiredoxin (Prx) or GSSG to GSH. NADPH is over-produced during the reprogramming of energy metabolism, mainly through the pentose phosphate pathway (PPP), activated in the Warburg effect [49]. Therefore, HPV-negative HNSCC could cope with high levels of ROS induced by IR due to the activation of their antioxidant battery and PPP activation via the Warburg effect, which does not occur in HPV-positive HNSCC [44,45,50].

## 4. Nuclear Factor (Erythoid-Derived 2)-Like 2 (Nrf2) Induces Radioresistance in HNSCC

Cells produce a myriad of RONS and free radicals, which are defined as chemical species that exist independently and contain one or more unpaired electrons. Those molecules are produced either by endogenous processes such as electron leakage in the electron transport chain (ETC) of the mitochondria or exogenous processes (smoking, ozone exposure, hyperoxia, IR or heavy metal ions), which can damage DNA, lipids, proteins or carbohydrates causing cellular dysfunction and/or cell death [51]. Cells have developed antioxidant systems that regulate these oxidant species; however, if such systems are overcome, OS is induced [52]. Therefore, efficient mechanisms for the protection against oxidant metabolites are necessary to maintain cell viability. These include both, antioxidant enzymes and non-enzymatic systems that use chelators or scavengers of ROS [53]. For example, vitamin C (ascorbic acid), vitamin E (α-tocopherol), GSH, carotenoids (β-carotene) and ubiquinone are all low-molecular weight antioxidants that neutralize free radicals, preventing cell damage from OS [54,55]. On the other hand, enzymes with antioxidant activity also prevent cell damage caused by ROS. Such is the case of SODs (Cu-Zn SOD and Mn-SOD), Prxs, GPx, glutathione S-transferases (GST), CAT and heme oxygenase-1 (HO-1), which are activated in response to OS. SODs, Prxs, GPx, GST, CAT and HO-1 are regulated by the transcription factor Nrf2 [56,57]. Nrf2 is one of the main transcription factors involved in the antioxidant response, since under OS, it dissociates from the Keap1/Cul3 complex avoiding its degradation via proteasome, which in turn stabilizes Nrf2, which is now able to translocate to the nucleus [57]. In the nucleus Nrf2 binds to the antioxidant response elements (ARE) of genes encoding antioxidant enzymes, as well as phase II detoxification enzymes of rapid induction and activation [56]. The main genes activated by Nrf2 include antioxidant enzymes such as HO-1 [58,59,60], NAD(P) H:quinone oxidoreductase 1 (NQO1) [60,61] and Prx1 [62,63]. It has been shown that in prostate cancer Nrf2 activation protects against OS, inducing radioresistance [64]. On the other hand, loss of Nrf2 activation is associated with radiosensitivity in lung and pancreatic cancers [61,65]. It has been demonstrated that in HPV-negative HNSCC cells, activation of Nrf2 is also associated with resistance to treatment [66] and conversely, its deactivation induces radiosensitivity. This Nrf2 activation in HNSCC is also associated to the upregulation of HO-1, NQO1 and GST, which are effective antioxidants (Figure 1) [67]. 

On the contrary, it has been shown that the transfection of HPV E6/E7 oncogenes in HNSCC cells induce ROS production via NADPH oxidase 2 (Nox2), another source of ROS generation that does not involves electron leakage in the mitochondrial ETC, which increases radiosensitivity in HPV-positive HNSCC (Figure 1) [46].

## 5. Radioresistance and Glucose Metabolism in HNSCC

The reprogramming of energy metabolism, considered as a hallmark of cancer, is a dynamic process which is induced to ensure a stable generation of energy and biomass, where glucose is the major molecule that supplies both [68,69]. In differentiated cells, glucose is metabolized through glycolysis and OXPHOS, where glycolysis, carried out in the cytosol, metabolizes glucose to pyruvate, which in turn is transported into the mitochondria and degraded to acetyl-CoA, continuing its metabolism in the tricarboxylic acid (TCA) cycle, producing flavin adenine dinucleotide (FADH_2_), guanosine triphosphate (GTP), coenzyme A (Co-A-SH) and nicotinamide adenine dinucleotide (NADH). NADH functions as an electron donor promoting OXPHOS. Therefore, the major amount of energy in the form of ATP is obtained in the mitochondria, since in OXPHOS, 34 ATP molecules are obtained in comparison with 2 ATP obtained by in glycolysis in the cytosol. However, in cancer cells glucose is converted to lactate instead of acetyl-CoA from pyruvate, even when mitochondria are functional in the presence of an adequate oxygen supply and OXPHOS can be carried out. The process where glycolysis is favoured instead of OXPHOS, yet with regular O_2_ concentrations in cancer cells, is called Warburg effect [27]. This process is related to elevated levels of lactate and pyruvate, due to the overexpression and activation of glycolytic regulatory enzymes, such as hexokinase (HK), phosphofructokinase (PFK) and pyruvate kinase (PK) [70,71,72]. Moreover, *ras*, *src* and *myc* oncogenes induce the overexpression of glucose transporters (GLUT1 and 3), providing glucose and maintaining the Warburg effect [73,74]. 

**Figure 2:**
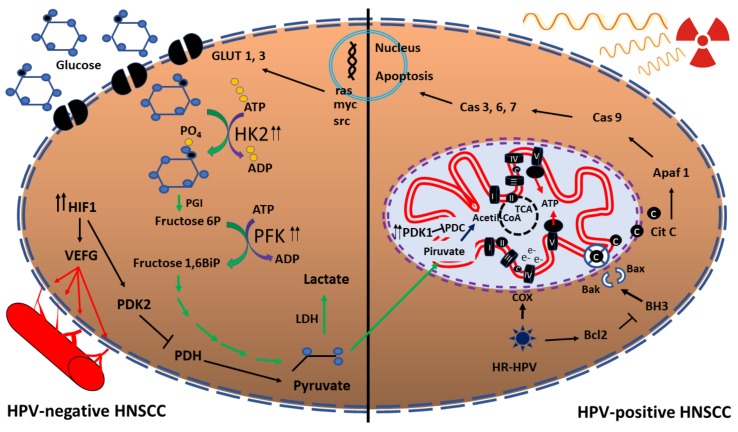
Metabolic reprogramming in HNSCC in response to IR. Activation of glycolysis and HIF1 in HPV-negative HNSCC cells induce radioresistance, in comparison to radiosensitive HPV-positive HNSCC cells, where OXPHOS is activated increasing ROS production and OS. In the presences of HPV, COX augments, activating the electron transport chain. Glucose transporter (GLUT), hexokinase (HK), phosphoglucose isomerase (PGI) phosphofructokinase (PFK), hypoxia-inducible factor (HIF), vascular endothelial growth factor (VEFG), pyruvate dehydrogenase kinase (PDK), pyruvate dehydrogenase (PDH), pyruvate dehydrogenase complex (PDC), cytochrome c oxidase (COX), tricarboxylic acid (TCA) cycle, cytochrome (cit), apoptosis protease-activating factor (Apaf), caspase (Cas), lactate dehydrogenase (LDH). The double arrow represents overexpression.

The Warburg effect gives a metabolic advantage to cancer cells in order to produce fast energy and biomass, as well as glycolytic intermediates, which can be used in other processes, such as the PPP, lipid synthesis or nucleotide production [27]. Moreover, this metabolic rewiring causes resistance to radiotherapy in cancer cells. Kunkel et al. [75] demonstrated that GLUT-1 is overexpressed in radioresistant HNSCC tumours; however, if this transporter is inhibited, HNSCC are sensitized to IR [76]. The increase of GLUT-1 was associated with an increase of glucose uptake in radioresistant HNSCC cells, where glucose metabolism is favoured in comparison with glutamine metabolism [77]. HPV-negative HNSCC cells promote glycolysis, where an overexpression of HK2 and PDK1 enzymes occurs [78]. It was demonstrated that when an inhibitor of PDK1 was used in HPV-negative HNSCC, the cells became radiosensitized, which supports the association of glucose metabolism and HNSCC radioresistance [78]. In contrast, HPV-positive HNSCC cells express low levels of HK2 and PDK1 enzymes (Figure 2). 

## 6. Role of Mitochondria in Irradiated HNSCC

Mithochondria are closely related to tumorigenesis, where multiple processes are undertaken such as OXPHOS, fatty acid oxidation (β-oxidation) or aspects involving mitochondrial fission, fusion and biogenesis, as well as cell signaling and cell death [79]. Therefore, the participation of mitochondria in cancer is critical, permitting cancer cells to adapt to cellular metabolic requirements. Moreover, the mitochondria have a critical role in cancer treatments including radiotherapy [80]. Li et al. [81] showed that in HNSCC cells, growth differentiation factor 15 (GDF15) promotes radioresistance, activating mitochondrial membrane potential and decreasing ROS through the SMAD1 pathway. Although radiotherapy induces ROS, cancer cells can decrease ROS production through the increase of antioxidants such as Mn-SOD, causing radioresistance [82]. As a counterpart, it has been shown that in a nasopharyngeal carcinoma cell model the silencing of Mn-SOD gene induces radiosensitivity [83]. 

It has been demonstrated that HPV-positive HNSCC cells use mitochondrial respiration instead of glucose metabolism, since high levels of cytochrome c oxidase (COX), the key enzyme in the mitochondrial respiratory pathway, are produced, with a significative increase in the COX/HKII ratio [78]. In comparison, in HPV-negative HNSCC the mitochondrial OXPHOS is decreased, favouring the glycolytic process (Figure 2) [77]. 

Mitochondria also function as a checkpoint for proliferation, since they sense the cell suitability before this demanding metabolic process starts. Furthermore, mitochondria can release cytochrome c, inducing apoptosis; however, BCL2 and BCL2 like 1 (BCL2L1), that are antiapoptotic proteins, are overexpressed in HNSCC, which avoids cytochrome c release [84], inducing radioresistance (Figure 2) [84,85,86,87]. 

## 7. Hypoxia and ROS in Response to Radiotherapy in HNSCC

Oxygen is the key molecule in the respiration process, because it receives the electrons, producing water in the last step of OXPHOS [88]. Nevertheless, cells can be deprived of a constant feed of oxygen, in a process known as hypoxia, favouring the Warburg effect and angiogenesis [89,90]. Hypoxia activates different transcription factors such as the members of the HIF family: HIF1, HIF2 and HIF3, which in turn induce the expression of the vascular endothelial growth factor (VEFG), a key transcription factor that favours angiogenesis and facilitates the assimilation of oxygen and nutrients [91]. HIF1 activation is associated to radioresistance, since it activates different genes associated to cell cycle arrest such as MYC or CDC25A [92,93] and glycolysis through the upregulation of pyruvate dehydrogenase kinase 2 (PDK2), decreasing pyruvate dehydrogenase (PDH) activity. In consequence, pyruvate levels augment avoiding its processing to acetyl-CoA [94]. It has been shown that HIF1 is overexpressed in HPV-negative HNSCC cells, but it is downregulated in HPV-positive HNC (Figure 2) [78].

## 8. Lipid Metabolism in HNSCC Radioresistance

Cancer cells can reprogram lipid metabolism, including the increase in the uptake of exogenous lipids or the constant synthesis of monounsaturated fatty acids (MUFAs) and polyunsaturated fatty acids (PUFAs), in order to satisfy the high demand of biomass and energy [95]. Moreover, long-chain polyunsaturated fatty acids (LC-PUFAs), such as arachidonic acid (AA), eicosapentaenoic acid (EPA) and docosahexaenoic acid (DHA), are related to a wide variety of biological functions, since LC-PUFAs are substrates of eicosanoids that work as signal molecules or transcription regulators [96]. PUFAs are also major components of membrane phospholipids, affecting their permeability, transport functions, ion channel formation and fluidity, which depends on the number and type of these fatty acids [97]. A decrease in these lipids favouring a membrane rich in saturated phospholipids protects from lipid peroxidation and decrease antitumor drug uptake, increasing their resistance to treatment [98]. However, cancer cells can interchange saturated for polyunsaturated fatty acids to induce motility and metastasis [95]. Through both sources, uptake and synthesis can induce lipid accumulation, which is stored in lipid droplets (LDs); so, in cancer cells, the LDs increase is considered as a hallmark of cancer aggressiveness, inducing resistance to chemotherapy [99]. 

Cells exposed to IR spread energy through water radiolysis inducing oxidants such as O_2_^.-^, H_2_O_2_, HO^.^, carbon, oxygen, sulphur, and nitrogen radicals. These molecules oxidize fatty acids, which then induce lipid peroxidation chain reactions, altering the structure and function of the cell. For example, lipid peroxidation produces mutagenic and carcinogenic by-products such as trans-4-hydroxy-2-nonenal (4HNE) and malondialdehyde (MDA) [13]. It was shown that MDA directly forms adducts with the pyrimidines and purines bases of DNA, generating mutations and DNA damage, that induce cell death when not repaired [47]. IR also induces changes in the plasma and organelles membranes, showing new activity and distribution of the membrane microdomains, which are clusters of fatty acids with ceramide and cholesterol. These microdomains are organizers of the intracellular transport and signaling events, which could trigger different cancer mechanisms such as migration or cell proliferation [100]. Although the IR dose and the composition of microdomains have a critical role in the induction of cell death or cell proliferation, the mechanism is not completely understood, needing further investigation. 

Mims et al. [77] showed that HPV-negative HNSCC radioresistant cells prefer to use glycolysis instead of OXPHOS to produce energy. This cancer hallmark could be detrimental in the inner part of tumours where the microenvironment is hypoxic, since glucose uptake and glycolysis significantly decrease [101]. However, these cells have increased fatty acid biosynthesis via fatty acid synthase (FAS) overexpression to produce ATP [68]. This advantage in HPV-negative HNSCC radioresistant cells could cover the energy requirement of the cancer cells for continued proliferation, avoiding cell death [77,101]. Therefore, lipid metabolism is also a critical process to be considered in the treatment of HNSCC associated to radiotherapy. 

The peroxidation of PUFAs in the cytoplasmic and mitochondrial membranes can induce programmed cell death (apoptosis and/or autophagy). Thus, after exposure to radiation, cells need to cope to the process of peroxidation by reprogramming the metabolism of lipids and/or by inducing a battery of antioxidants that protect from lipid peroxidation [13]. These include SODs, catalase, peroxidases and vitamin E, among others. Therefore, oxidative effects in the cell due to radiation exposure are decreased by the increase in antioxidant levels and through the changes in metabolic pathways. However, these protective mechanisms could not be sufficient to deal with OS, inducing apoptosis and/or autophagy. 

## 9. Autophagy in HNSCC Radioresistance

Autophagy is a programmed cell death process that involves cellular recycling using the degradation of cellular constituents in lysosomes. Autophagy also maintains homeostasis providing cell cytoprotection, which involves remodeling, recovery, and renewal of the cells, removing cell damage and hazardous aggregates of proteins, as well as whole organelles such as mitochondria, or intracellular pathogens [102]. It has been shown that nuclear tumour zones have deficient vascularization, inducing nutrient and oxygen deprivation and metabolic stress. In these conditions, autophagy is triggered promoting the metabolism of cellular proteins, carbohydrates and lipids in order to provide nutrients, which avoids metabolic stress and in consequence maintains cell survival and tumour growth [103]. In these tumour zones, the mammalian target of rapamycin (mTOR) is deactivated, which induces the autophagy process; conversely, autophagy deactivation is due to the reactivation of mTOR, so there exists an inversely proportional relationship between autophagy and mTOR [104]. Therefore, autophagy has a significant role in feeding carbon metabolism, including carbohydrates, lipids, and nucleotides. These molecules that proceed from autophagy can enter and reinforce glycolysis, TCA cycle activity or PPP pathway [105]. In this way, cancer cells have an incredible adaptation to nutrient deprivation rewiring carbon metabolism through the autophagy process. 

It has been proposed that autophagy, but not apoptosis, is the main contributor to radioresistance, eliminating cell damage and contributing to carbon metabolism [106]. Autophagy is activated via p63, which is overexpressed in HNSCC radioresistance [107,108]. Regarding HPV, in cells that express E7, autophagy is increased providing molecules to maintain carbon metabolism [109]; but in contrast, E6 expression activates mTOR, the principal deactivator of autophagy [110]. Since in cancer, both E6 and E7 oncoproteins are present, the ratio of expression of E6/E7 could determine the induction of autophagy. However, to the best of our knowledge HPV-positive HNSCC autophagy induced by IR has not yet investigated.

Although HPV-negative or -positive HNSCC autophagy is a critical cellular process in response to IR, it has been poorly explored, needing further studies in order to provide a new strategy to treat HNSCC radioresistance. 

## 10. Metabolic Therapy for HNSCC

Tumour metabolism has been considered as a potential therapeutic target. Both normal proliferating cells and cancer cells, through the Warburg effect, can metabolize nutrients and produce ATP less efficiently than by OXPHOS, regardless of the presence of oxygen. Since cancer cells have an increased proliferative rate, they increase glucose intake to compensate their energy needs [27]. It has been demonstrated that if the Warburg effect is blocked, the death of cancer cells is selectively promoted [49]. Now it is known that most types of cancer contain metabolically functional mitochondria, which have critical function in tumorigenesis [111,112,113].

There are clear differences between HPV-positive and HPV-negative HNSCC. Moreover, it has been shown that HPV-positive HNSCC has a better prognosis, associated with a better response to radiation treatment, with a 5-year survival rate of 80%, in comparison with the 50% survival rate of HPV-negative HNSCC [3,114]. This contrast has allowed recognizing HPV-positive and HPV-negative HNSCC as two distinct varieties. Despite this, both HNSCC are treated similarly, consisting mainly in surgery with concomitant radio and/or chemotherapy [115,116]. The scope of these standard strategies is limited, since more than 50% of the patients present recurrence, which is associated with the inherent resistance to treatment of HNSCC [117]. The toxicity due to the treatment represents an important issue, so research has focused on new therapeutic strategies to improve outcomes, especially in HPV-negative patients, as well as on a decrease in the intensity of standard therapy for HPV-positive patients. 

HPV screening in HNSCC will help to choose a more appropriate therapy according to the characteristics of the tumour, trying to avoid resistance to treatment. For example, oxygen therapy where haemoglobin is increased [118], could be more efficient in HPV-positive HNSCC because these cancers induce ROS in different ways, which could synergize with ROS induced by oxygen and radiation therapy. The use of anti-cancer drugs directed to mitochondria (mitocans) is another possibility to treat HPV-positive HNC. For instance, metmorfin/phenformin, which besides decreasing blood glucose, insulin levels and PI3K activation, also inhibits complex I of ETC and intermediates of TCA in cancer cells [119,120]. Moreover, the VLX600 drug is an ETC inhibitor, which induces a significant reduction of colon cancer tumour growth [121]. It has also been shown that the use of non-selective beta-blockers such as propranolol displays significant anti-cancer effects in a murine model of recurrent/metastatic HPV-positive HNSCC. VLX600 shows a significant reduction in tumour cell mitochondrial metabolism, which together with dichloroacetate (DCA), a clinically available glycolytic inhibitor, has proved to be an effective therapy against HNSCC in murine models, since the VLX600–DCA combination dramatically attenuates tumour cell metabolism and mTOR signaling [122,123]. This novel combination enhances the effects of standard treatments [124].

On the other hand, since HPV-negative HNSCC increase the glycolytic pathway, anti-glycolytic drugs administered concomitantly to chemotherapy could increase the efficacy in the antitumoral response. For example, tyrosine kinase (TK) inhibitors, such as imatinib, decrease glycolysis, affecting glycolytic enzymes such as HK and PFK-1 via HIF-1 [125]. Moreover, inhibition of fatty acid synthesis, by blocking fatty acid synthase with different drugs, such as orlistat [77], could be another way to improve treatment response in HPV-negative HNSCC. 

Metabolic therapies could have a significant importance in the elimination of cancer cells, since they target the metabolites used to produce energy and cell biomass. Since cancer cells express a particular set of enzymes, these enzymes could be targeted in order to specifically eliminate cancer cells, minimizing injury in normal cells. Therefore, several preclinical and/or clinical studies are being carried out using different compounds that act on metabolic pathways. Such is the case of metformin, which inhibits mitochondrial complex I and the synthesis of lipids and proteins. Metformin, which has been used to treat type II diabetes for over 50 years, has the property of slowing down cancer growth [126,127]. Essentially, since the growth of cancer cells is accelerated in low oxygen concentrations, metformin improves oxygen concentration in cancer tissue, inducing OS and cancer cell death via apoptosis [128]. In this direction, a clinical study that is now being carried out, intends to verify the effect of this drug in the oxygen content in HNC, and to evaluate whether changes in the oxygen content within the tumour can be visualized by means of magnetic resonance imaging (clinicaltrials.gov no NCT03510390). 

Preclinical studies showed that lonidamine targets glycolysis through the inhibition of HEKII [129], which increases the expression of the apoptosis-associated proteins Poly (ADP-ribose) polymerase (PARP) and caspase 3, inducing radiosensitization [130,131]. It has been shown that, at concentrations that are safe for humans, lonidamine impedes energy-dependent repair of potentially lethal damage induced by radiation [132]. Previous studies demonstrated that the combination of hyperfractionated radiotherapy and lonidamine increased the proportion of long-term disease-free patients [133].

Dichloroacetate, which inhibits the production and transport of lactate, has been tested for metabolic therapy. A combination of this drug with cisplatin and radiation is already running in a HNSCC clinical trial stage III-IV (NCT01386632). Targeting energetic metabolism as an anti-cancer therapy is a promise for HNSCC treatment. Nevertheless, differences between HPV-positive and -negative HNSCCs cases should be taken into account, in order to use the correct metabolic treatment to obtain the best response.

## 11. Conclusions

Reprogramming of energy metabolism is an important hallmark in cancer development, which permits cancer cells to continue growing, even under stress conditions such as hypoxia, where nutrients and oxygen are deprived. This hallmark can provide protection to IR in cancer cells. It has been shown that HPV-negative HNSCC tumours induce the reprogramming of energy metabolism, activating the Warburg effect and lipid metabolism in comparison with HPV-positive HNSCC, which favours OXPHOS to obtain ATP. Such metabolic difference in HPV-negative HNSCC coupled with the status of p53, a better capacity of DNA repair and better coping with OS, induce an undesirable response to IR, avoiding cell death and promoting radioresistance, in comparison with HPV-positive HNSCC. However, a small percentage (20%) of HPV-positive HNSCC does not respond properly to treatment, which suggests the necessity of new strategies to cope with this issue. The design of an appropriate therapy to target glycolysis, lipid metabolism or mitochondrial metabolism, associated to HPV status, could improve the response to treatment in HNSCC.
